# Optimization of Black Garlic Protein Extraction Process and Exploration of Its Properties and Functions with Enzymatic Hydrolysis Products

**DOI:** 10.3390/molecules30010125

**Published:** 2024-12-31

**Authors:** Jian Liu, Yuanyuan Wang, Bo Wang, Wei Zhang, Xiaoyu Ren, Youchuang Zhang, Lijun Jiang, Chunming Dong, Guihong Zhao

**Affiliations:** 1 College of Agriculture and Bioengineering, Heze University, Heze 274000, China; liujianscholar@163.com (J.L.); wyy4461@163.com (Y.W.); caoliwuxi@163.com (B.W.); 18353585560@163.com (X.R.); 17263360294@163.com (Y.Z.); lijunjiang9019@hotmail.com (L.J.); 2 Institute of Biotechnology, Chinese Academy of Tropical Agricultural Sciences, Haikou 570100, China; 3 College of Marine and Environmental Sciences, Tianjin University of Science and Technology, Tianjin 300457, China; 4 Inspection and Testing Center of Linshu County, Linyi 276700, China; 18005399698@163.com

**Keywords:** black garlic, extraction process optimization, functional properties, in vitro biological activity

## Abstract

This study optimized the process of extracting protein from black garlic using an alkaline dissolution and acid precipitation method through response surface methodology. The optimal extraction conditions were determined as a solid-to-liquid ratio of 1:50, an extraction time of 100 min, an extraction temperature of 30 °C, and an alkaline extraction pH of 9.0. Under these optimized conditions, the actual black garlic protein (BGP) extraction yield was 12.10% ± 0.21%, and the isoelectric point of the obtained BGP was 3.1. Subsequently, this study extracted black garlic protein under optimal conditions and subjected it to enzymatic hydrolysis using different enzymes (trypsin, pepsin, and their mixed enzymes). The functional characteristics, antioxidant activity, and hypoglycemic activity of black garlic protein before and after enzymatic hydrolysis were compared. Among the hydrolysates, the pepsin hydrolysate (BGPH-P) had the smallest particle size (188.57 ± 1.93 nm) and the highest Zeta potential (−29.93 ± 0.42 mV). Scanning electron microscopy showed that BGPH-P had the smallest and most dispersed particles. Fourier-transform infrared (FTIR) spectroscopy revealed that the dual enzymatic hydrolysis hydrolysate (BGPH-PT) exhibited the most stable structure. Compared to BGP, the hydrolysates demonstrated significantly improved solubility, water-holding capacity, and foaming ability (*p* < 0.05), while their emulsifying activity, emulsion stability, DPPH radical scavenging capacity, and hypoglycemic activity decreased. In summary, the BGP extracted using the optimized process demonstrated good antioxidant and hypoglycemic activities, while its enzymatic hydrolysate BGPH-P exhibited excellent solubility, water-holding capacity, and emulsifying properties, providing valuable insights for the further development of black garlic protein and its hydrolysates.

## 1. Introduction

With the development of the health food industry, black garlic is becoming increasingly popular as a functional food, which is produced by pretreating, aging, and drying fresh garlic under strictly controlled temperature and humidity conditions, and undergoing Maillard reaction during this process [1]. Black garlic is rich in proteins, polysaccharides, vitamins, polyphenols, amino acids, sulfur-containing compounds, organic acids, flavonoids, and carotenoids, as well as various minerals and trace elements [2]. These components provide black garlic with both its unique flavor and nutritional value, while also exhibiting significant biological activities, such as antioxidant, anti-inflammatory, immune-regulatory, cardiovascular protective, and anticancer effects [3]. Currently, black garlic is sold in the form of primary products, including fermented garlic and black garlic powder, which limits the full utilization of its functional components and results in the loss of its potential nutritional value [4]. Hence, more effective methods for processing and extracting active ingredients from black garlic are urgently needed to fully exploit its nutritional and health benefits. This will help promote the wider application of black garlic in the functional food market and further facilitate its development and promotion as a key nutritional resource.

In recent years, plant-based proteins and their hydrolysates have emerged as a research hotspot in food science and nutrition due to their excellent nutritional value and diverse health benefits. Compared to animal-derived proteins, plant-based proteins provide higher digestibility, lower allergenicity, and reduced cholesterol levels, while their production processes have a lower environmental impact, aligning with sustainable development trends. Li et al. [5] found that quinoa protein extract effectively improved DSS-induced colitis in mice by regulating the gut microbiota and inhibiting inflammatory responses, contributing to the treatment of intestinal inflammation. Paterson et al. [6] explored the multifunctionality of soy proteins in immune cell models, revealing that soy proteins regulate immune responses and exhibit anti-inflammatory, antioxidant, and immunomodulatory activities. Protein hydrolysates are the products obtained by breaking down large proteins into smaller peptides and free amino acids through enzymatic or chemical methods [7]. This process is known as proteolysis, and commonly used enzymes include pepsin, trypsin, and alkaline protease. The primary characteristics of protein hydrolysates are their smaller molecular weight, ease of absorption, and their tendency to exhibit biological activities that differ from those of the original proteins. Manzoor et al. extracted protein hydrolysates from apple seeds and found that they effectively inhibited the in vitro activity of α-glucosidase, pancreatic lipase, and angiotensin-converting enzymes [8]. Liu et al. extracted protein hydrolysates from mung beans and found that the enzymatic hydrolysis process significantly improved the solubility, emulsification, and foaming properties of mung bean protein [9]. Moreover, the hydrolysates exhibited strong free radical scavenging activity, indicating potential functional value in antioxidant applications. Currently, research on black garlic is primarily focused on its active components, including polyphenols, sulfur-containing compounds, polysaccharides, and amino acids [10]. In contrast, studies on black garlic proteins and their hydrolysates are relatively limited; more extensive research has been conducted on garlic proteins and their hydrolysates. Gao et al. extracted proteins and their hydrolysates from garlic, demonstrating that garlic proteins and hydrolysates exhibit significant antioxidant and antihypertensive effects by scavenging free radicals and inhibiting ACE activity [11]. Chidike Ezeorba et al. extracted proteins and peptides from garlic, showing that they possess antioxidant, anti-inflammatory, and immunomodulatory properties [12]. In addition, although the protein and other components of black garlic may undergo various transformations during fermentation, the characteristics of the protein and the enzymatic hydrolysates of black garlic still need to be further explored. Therefore, systematically investigating the structural characteristics and biological activities of black garlic proteins and their hydrolysates, particularly their antioxidant and hypoglycemic properties, could provide new insights into their potential applications in functional foods and pharmaceuticals.

Currently, the primary methods for extracting plant-based proteins include ammonium sulfate fractionation, organic solvent precipitation, ultrasound-assisted extraction, and alkali extraction coupled with acid precipitation [13]. Ammonium sulfate fractionation is suitable for the preliminary separation of proteins but involves complex procedures and yields relatively low protein purity. Organic solvent precipitation can effectively remove lipids and pigments, though it may damage protein structures. Ultrasound-assisted extraction demonstrates high efficiency but poses risks to protein stability. In contrast, alkali extraction coupled with acid precipitation has become the preferred method for black garlic protein extraction due to its high yield, simplicity, and low cost. By adjusting pH, proteins can be selectively separated while preserving their natural structure and bioactivity under mild conditions [14]. Optimizing the extraction conditions for black garlic protein using this method not only enhances protein yield and purity but also provides high-quality materials for further research on black garlic proteins and their hydrolysates. This approach is of significant theoretical and practical value for developing functional foods and high value-added products from black garlic.

In this study, the alkali-acid extraction method was systematically optimized through single-factor experiments and response surface methodology to determine the optimal extraction parameters for black garlic protein. Based on these optimizations, black garlic protein was hydrolyzed to prepare hydrolysis products, and subsequently the structural characteristics, physicochemical properties, and functional properties of the original protein were analyzed and characterized. This study not only helps to gain a deeper understanding of the characteristics and functions of protein, providing theoretical support for deep processing, but it can also expand the application of black garlic in functional foods, health products, and pharmaceuticals.

## 2. Results

### 2.1. Analysis of the Optimal Extraction Process for Black Garlic Protein

#### 2.1.1. Determination of the Isoelectric Point and Analysis of the Results

**Figure 1:**
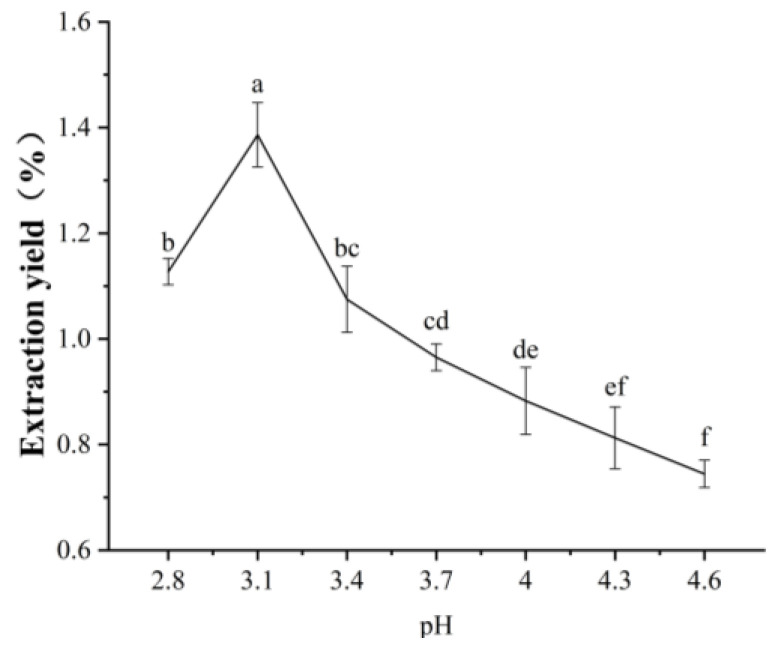
Extraction yield of black garlic protein at different pH. Different letters indicate significant differences (*p* < 0.05).

Isoelectric point (pI) refers to the specific pH at which a molecule’s net surface charge is zero, which is critical for understanding the behavior of zwitterions (like proteins) in different pH environments. In this study, black garlic protein was extracted using an alkali extraction followed by acid precipitation, involving the isoelectric point during the acid precipitation stage. To optimize the extraction process and determine the best extraction parameters, the pH of the solution was gradually adjusted to 2.8, 3.1, 3.4, 3.7, 4.0, 4.3, and 4.6, with sufficiently small pH differences between samples. The results showed that the protein yield peaked at pH 3.1 (Figure 1), indicating the protein had a net charge of zero at this pH, leading to minimal solubility and maximum precipitation. Therefore, the isoelectric point of black garlic protein was determined to be 3.1.

#### 2.1.2. Analysis of Single-Factor Results

**Figure 2:**
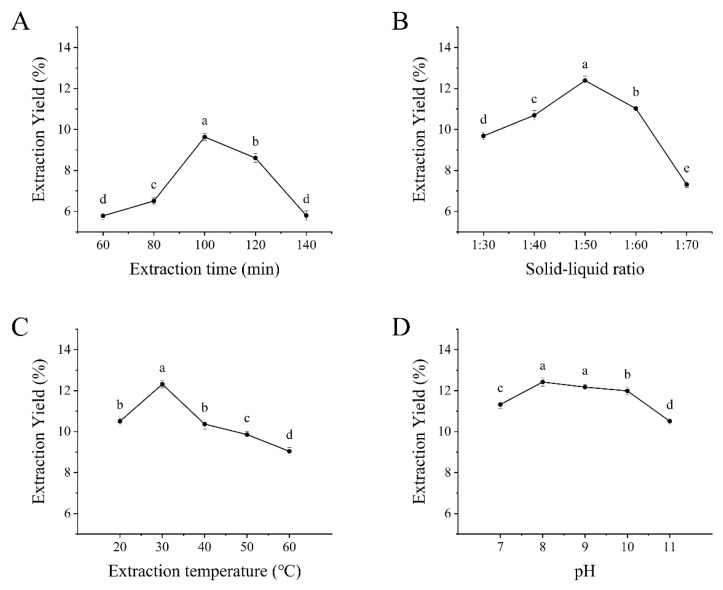
Results of single-factor experiments. (**A**) Effects of different extraction times on the extraction yield of black garlic protein. (**B**) Effects of different solid–liquid ratios on the extraction yield of black garlic protein. (**C**) Effects of different extraction temperatures on the extraction yield of black garlic protein. (**D**) Effects of different alkaline extraction pH values on the extraction yield of black garlic protein. Different letters indicate significant differences (*p* < 0.05).

After determining the isoelectric point of black garlic protein, the effects of various extraction conditions on black garlic protein yield were further investigated. The single-factor experiment results (Figure 2) demonstrated that extraction time, solid-to-liquid ratio, extraction temperature, and alkali extraction pH significantly affected the extraction yield of black garlic protein (*p* < 0.05). The extraction yield increased initially but decreased with extended extraction time. The highest yield (9.63%) was achieved at 100 min, likely because the black garlic powder was fully swelled in the solution, and then facilitated protein extraction [15]. The yield decrease beyond 100 min was possibly due to protein degradation or oxidation during prolonged extraction time (Figure 2A). Thus, 100 min was determined to be the most suitable extraction time for subsequent response surface methodology experiments.

As shown in Figure 2B, as the solid-to-liquid ratio increased, the extraction yield of black garlic protein initially rose and then declined. The highest yield (12.39%) was achieved at the solid-to-liquid ratio of 1:50. A related study showed that increasing the solvent volume (i.e., the solid-to-liquid ratio) can enhance the concentration gradient, facilitating protein diffusion from the solid phase to the liquid phase, thereby increasing the extraction yield [16]. However, an excessively high solid-to-liquid ratio may result in excessive solvent, causing a dilution effect that reduces protein concentration and extraction efficiency (Figure 2B). Therefore, a solid-to-liquid ratio of 1:50 (g/mL) was determined to be optimal for subsequent response surface methodology experiments.

On the basis of Figure 2C, as the temperature increased, the extraction yield of black garlic protein initially rose and then declined. The highest yield (12.39%) was achieved at 30 °C. Optimal temperature promotes the Brownian motion of protein molecules [17], facilitating protein dissolution and accelerating water molecule motion, thereby enhancing the extraction efficiency (Figure 2C). However, temperatures above 30 °C may cause protein denaturation, reducing solubility and lowering extraction yield. So, 30 °C was determined to be the optimal extraction temperature for subsequent response surface methodology experiments.

Finally, the extraction yield rapidly increased as the alkali extraction pH rose from 7 to 8, peaking at 12.39% when the pH was at 8. As the pH increased to 9, 10, and 11, the extraction yield slightly declined (Figure 2D). This may be attributed to the fact that increasing the distance from the isoelectric point enhances electrostatic repulsion between protein molecules, which in turn reduces aggregation and consequently improves their solubility in the solvent [18]. Furthermore, the protein solubility was relatively high at higher pH levels, and the excessively alkaline environment may disrupt protein structure, thereby reducing stability and leading to a decrease in extraction yield. Hence, a pH of 8 was determined to be optimal for subsequent response surface methodology experiments.

#### 2.1.3. Response Surface Analysis Results

Based on the results of single-factor experiments, the following parameters were selected to optimize the extraction conditions: extraction time (80, 100, 120 min), solid-to-liquid ratio (1:40, 1:50, 1:60 g/mL), extraction temperature (20 °C, 30 °C, 40 °C), and extraction pH (8, 9, 10). As shown in Table 1, a Box–Behnken response surface design was used, and multivariate regression analysis was performed using Design-Expert 12 software. A quadratic regression equation was established with black garlic protein yield (Y) as the dependent variable, and extraction time (A), solid-to-liquid ratio (B), extraction temperature (C), and alkali extraction pH (D) as independent variables:

Y = 122.40 + 0.36A + 0.80B + 0.73C + 1.06D − 4.00AB + 2.15AC + 2.07AD + 1.85BC − 1.05BD + 6.40CD − 4.07A^2^ − 5.93B^2^ − 5.63C^2^ − 7.47D^2^.

**Figure 3:**
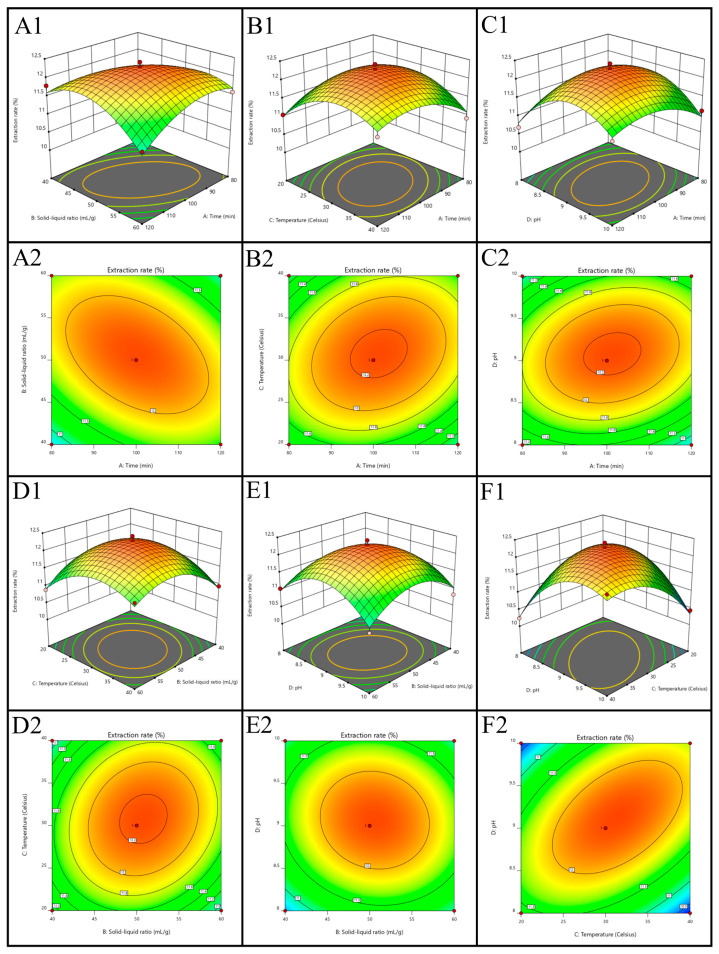
Response surface diagram shows the influence of factor interaction on the protein extraction yield of black garlic. (**A1**,**A2**) represent the 3D surface plot and contour plot for the interaction between extraction time and solid-liquid ratio, respectively. (**B1**,**B2**) represent the 3D surface plot and contour plot for the interaction between extraction time and extraction temperature, respectively. (**C1**,**C2**) represent the 3D surface plot and contour plot for the interaction between alkali extraction time and alkali extraction pH, respectively. (**D1**,**D2**) represent the 3D surface plot and contour plot for the interaction between extraction temperature and solid-liquid ratio, respectively. (**E1**,**E2**) represent the 3D surface plot and contour plot for the interaction between solid-liquid ratio and alkali solution pH, respectively. (**F1**,**F2**) represent the 3D surface plot and contour plot for the interaction between alkali solution pH and extraction temperature, respectively. Warm colors (red/yellow) indicate a higher extraction rate, while cool colors (green/blue) indicate a lower extraction rate.

The steepness of the response surface reflects the significance of the interaction between two factors: the steeper the surface, the stronger the interaction effect. Elliptical contour plots indicate a more pronounced interaction between two factors. Comparing the response surface and contour plots revealed that the interaction between extraction temperature and alkali extraction pH had the most significant effect on black garlic protein extraction, followed by the interaction between solid-to-liquid ratio and extraction time (Figure 3). The results of the analysis of variance (ANOVA) revealed that the *p*-value of the regression model was less than 0.0001, indicating a high level of significance and confirming that the model accurately reflected the relationship between extraction yield and the factors involved. The lack-of-fit *p*-value was 0.1303, greater than 0.05, indicating a good fit between the model and the experimental data. The multiple correlation coefficient (R^2^) was 0.9652, indicating a high degree of fit between the observed and predicted values (Table 2). Based on the F-values, the factors were ranked in terms of their influence as follows: alkali extraction pH (D) > solid-to-liquid ratio (B) > extraction temperature (C) > extraction time (A). In conclusion, the regression model can guide extraction experiments for black garlic protein and provide a theoretical basis for further optimization of the extraction conditions.

#### 2.1.4. Validation of the Response Surface Optimization Model

After evaluating the response surface regression model, the optimal conditions for black garlic protein extraction were determined. To simplify the experimental process, the adjusted extraction parameters were set as follows: a solid-to-liquid ratio of 1:50, extraction time of 100 min, extraction temperature of 30 °C, and alkali extraction pH of 9.0. Based on these parameters, three replicate experiments were conducted to verify the reliability of the model. The experimental results showed that the actual extraction yield of black garlic protein was 12.10 ± 0.21%, closely matching the model’s predicted value. This confirms that the response surface regression model is capable of effectively predicting the extraction efficiency of black garlic protein.

### 2.2. The Spectra and Morphology Analysis of Black Garlic Protein and Its Hydrolysates

#### 2.2.1. Particle Size and Zeta Potential Analysis

Particle size is crucial for understanding the bioactivity, stability, and solubility of proteins and their hydrolysates. Generally, smaller particle sizes are associated with improved solubility, emulsifying ability, and foaming capacity [19]. As shown in Table 3, the average particle sizes of BGP and its hydrolysates (BGPH-T, BGPH-P, and BGPH-PT) were 200.07 ± 2.86, 193.73 ± 0.69, 188.57 ± 1.93, and 191.20 ± 0.65 nm, respectively. The data indicated that the hydrolysates (BGPH-T, BGPH-P, and BGPH-PT) had smaller average particle sizes compared to the original BGP, with BGPH-P (pepsin-treated) having the smallest size, followed by BGPH-PT from dual enzymatic hydrolysis. This difference can be attributed to the partial degradation of the protein by pepsin, which generates smaller peptides while retaining certain secondary structures, particularly hydrophobic regions that were not fully exposed. Subsequent trypsin hydrolysis may have exposed additional hydrophobic regions, leading to their reaggregation through hydrophobic interactions and ultimately forming larger aggregates [20,21]. Therefore, the particle size of BGPH-PT was slightly larger than that of BGPH-P.

Zeta potential serves as a crucial indicator of protein stability in solution, where its absolute value provides insight into the magnitude of electrostatic repulsion forces acting between protein particles. A larger absolute Zeta potential signifies a greater degree of electrostatic repulsion, which serves to hinder aggregation and thereby enhances the stability of the solution. Conversely, a lower absolute Zeta potential implies that particles are more susceptible to aggregation, thereby decreasing the stability of the solution [22]. As shown in Table 3, the Zeta potentials of BGP, BGPH-T, BGPH-P, and BGPH-PT were −21.60 ± 1.85, −26.73 ± 1.00, −29.93 ± 0.42, and −29.73 ± 0.56 mV, respectively. BGPH-P exhibited the highest absolute Zeta potential, indicating the strongest electrostatic repulsion and highest stability in solution. The Zeta potential values followed this order: BGPH-P > BGPH-PT > BGPH-T > BGP. The hydrolysates had higher absolute Zeta potentials, indicating better dispersion and higher stability in solution. This finding deepens our understanding of the physical properties of hydrolysates and provides valuable insights for their potential applications in biological systems.

In conclusion, BGPH-P had the smallest particle size (188.57 ± 1.93 nm), suggesting superior solubility and dispersion in solution. Additionally, BGPH-P exhibited the highest absolute Zeta potential (−29.93 ± 0.42 mV), further indicating strong resistance to aggregation and enhanced stability in solution.

#### 2.2.2. Analysis of Scanning Electron Microscopy (SEM) Observation Results

**Figure 4:**
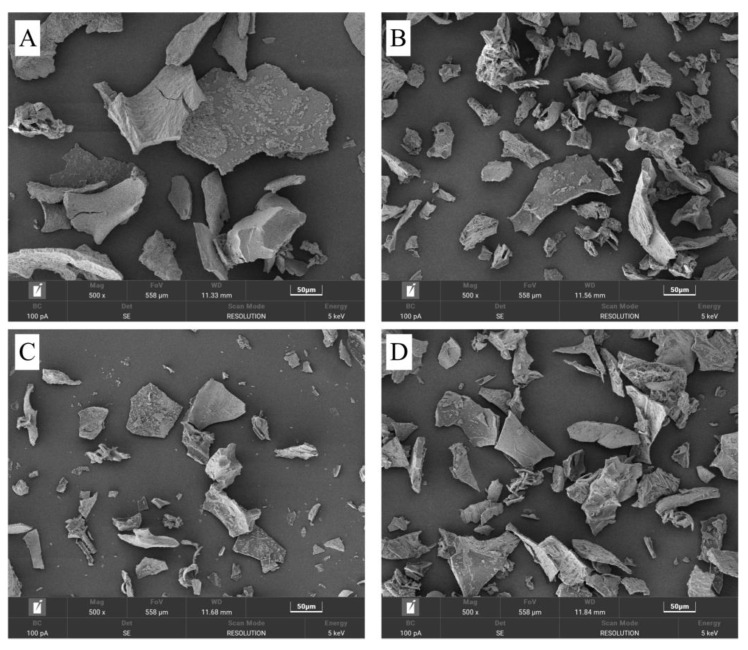
Apparent structure of black garlic protein and its hydrolysates determined by different enzymatic hydrolysis methods. (**A**) Apparent structure of BGP. (**B**) Apparent structure of BGPH-T. (**C**) Apparent structure of BGPH-P. (**D**) Apparent structure of BGPH-PT. All SEM images were obtained at 500×.

Scanning electron microscopy (SEM) is a powerful tool for visualizing surface structures, and it is widely applied in protein morphology analysis [23]. During the enzymatic hydrolysis of black garlic protein, enzymes like trypsin and pepsin cleave specific peptide bonds, disrupting the protein’s three-dimensional structure, leading to molecular fragmentation and altering its surface morphology. Figure 4 shows SEM images of BGP, BGPH-T, BGPH-P, and BGPH-PT samples at 500× magnification. The BGP sample exhibited larger particles with a dense, smooth surface, reflecting its intact molecular structure. In contrast, BGPH-T exhibited particles of smaller size with a rough surface texture and an abundance of pores, suggesting that trypsin-initiated hydrolysis occurred primarily at the surface, progressively cleaving internal peptide bonds and ultimately resulting in the formation of smaller, more uniform peptides. This observation aligns with Feng et al.’s [24] findings in trypsin-hydrolyzed walnut protein. BGPH-P also showed a rough surface, but was smoother with fewer pores compared to BGPH-T. This may be because pepsin hydrolyzes peptide bonds are positioned near aromatic amino acids, such as phenylalanine, tyrosine, and tryptophan, which are often located in the protein’s core regions [25]. While pepsin’s rapid degradation of these core regions can result in nearly complete protein degradation, it simultaneously prevents the formation of larger, rougher surface structures. Consistent with its smaller average particle size, the SEM observations of BGPH-P further confirm the strong impact of pepsin on protein structure. In addition, the BGPH-PT sample exhibited even smaller particles and a rougher surface, reflecting the deep structural changes from the synergistic action of both enzymes. These morphological changes likely enhance the protein’s functional properties, such as hydrophobicity and antioxidant activity. In summary, SEM analysis showed that BGPH-P had smaller particles, a smoother surface, and fewer pores, confirming the significant degradative effect of pepsin, which is consistent with its smaller average particle size. These structural changes may improve BGPH-P’s solubility, water-holding capacity, and emulsifying ability, thereby broadening its potential applications in food and other industries.

#### 2.2.3. Fourier Transform Infrared Spectroscopy Analysis

**Figure 5:**
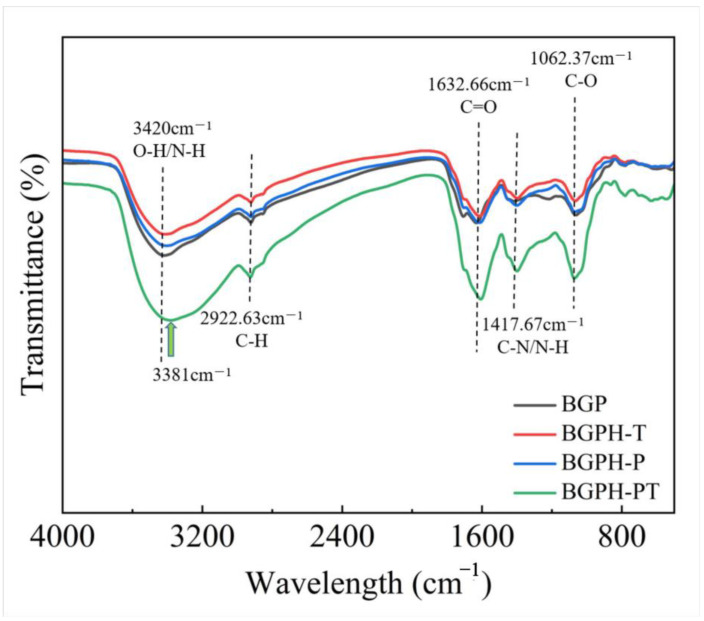
Fourier transform infrared spectrum of BGP, BGPH-T, BGPH-P, BGPH-PT.

Fourier Transform Infrared (FTIR) spectroscopy is a powerful tool for identifying specific spectral peaks, revealing protein structural characteristics, and is particularly useful for studying protein secondary structures [26]. As shown in Figure 5, black garlic protein (BGP) and its hydrolysates (BGPH-T, BGPH-P, BGPH-PT) exhibited characteristic infrared absorption bands, including the amide A (3300–3440 cm^−1^), amide B (3100−3200 cm^−1^), amide I (1600−1700 cm^−1^), amide II (1500−1600 cm^−1^), and amide III (1200−1350 cm^−1^) bands. These bands correspond to different vibrational modes of chemical bonds in proteins, with the amide I band, primarily associated with C=O stretching vibrations, being key for secondary structure analysis [27]. In the hydrolysates, the amide I band shifted to the right with increased peak intensity, likely due to enhanced hydrogen bonding and an increase in β-sheet structures. Additionally, the amide A band corresponds to the N-H stretching vibration in amino groups and the O-H stretching vibration in carboxyl groups [28,29,30]. Compared to the amide A absorption peak of black garlic protein (BGP) at 3420 cm^−1^, the hydrolysates (BGPH-T, BGPH-P, and BGPH-PT) show a right shift, with BGPH-PT displaying a notably enhanced peak intensity. This suggests the formation of more numerous or stronger hydrogen bonds in the hydrolysates. Enhanced hydrogen bonding restricts the N-H and O-H stretching vibrations, thereby reducing the vibrational frequency. Consequently, the absorption peak shifts to a lower wavenumber region (3381 cm^−1^). Thus, it can be inferred that the hydrolysates, especially BGPH-PT obtained a more stable structure after enzymatic hydrolysis.

### 2.3. Analysis of Functional Properties Results of Black Garlic Protein and Its Hydrolysates

#### 2.3.1. Solubility Determination

**Figure 6:**
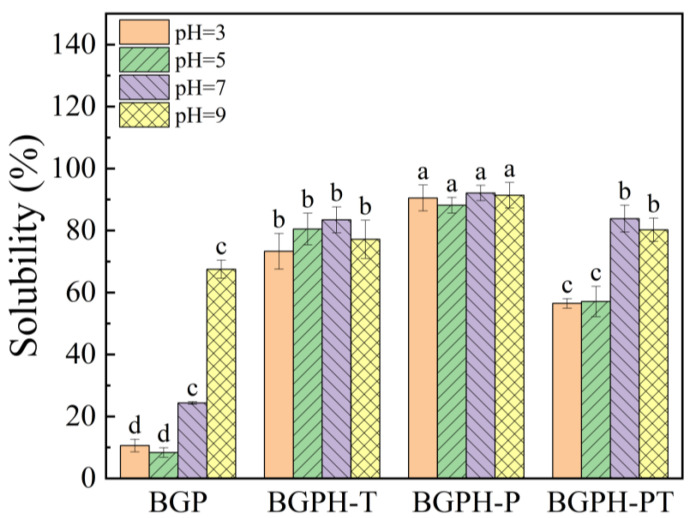
Results of solubility determination of BGP, BGPH-T, BGPH-P and BGPH-PT samples. Different letters marked in the same column indicate significant differences between groups (*p* < 0.05).

Protein solubility is a critical parameter for assessing its denaturation and aggregation, which plays a vital role in understanding its functional characteristics and potential applications [31]. As shown in Figure 6, this study explored the effects of various enzymatic hydrolysis methods on the solubility of black garlic protein (BGP). The results are as follows: Firstly, the solubility of untreated BGP is significantly influenced by the pH, with a decrease observed at lower pH values (pH 3 or 5). This is likely due to the proximity of these pH values to the isoelectric point, causing protein aggregation and precipitation. However, at pH 7 and 9, solubility significantly increases, likely because the alkaline environment enhances negative surface charges on the protein, increasing electrostatic repulsion and reducing molecular aggregation [32]. In addition, the solubility of BGPH-T and BGPH-P showed minimal variation across different pH levels. This can be attributed to the enzymatic hydrolysis that breaks down BGP into smaller peptides, which exhibit higher water solubility and maintain stability across a broad pH range. Finally, the pH exerts some influence over the solubility of BGPH-PT, with lower solubility observed at pH 3 and 5, and higher solubility at pH 7 and 9. This may be due to the formation of more complex peptide structures following dual enzymatic hydrolysis, which tend to aggregate in acidic conditions, thereby reducing solubility [33]. In summary, enzymatic hydrolysis can greatly enhance the solubility of BGP, with BGPH-P demonstrating superior solubility across all pH conditions, outperforming other samples.

#### 2.3.2. Water and Oil Holding Capacity Measurements

**Figure 7:**
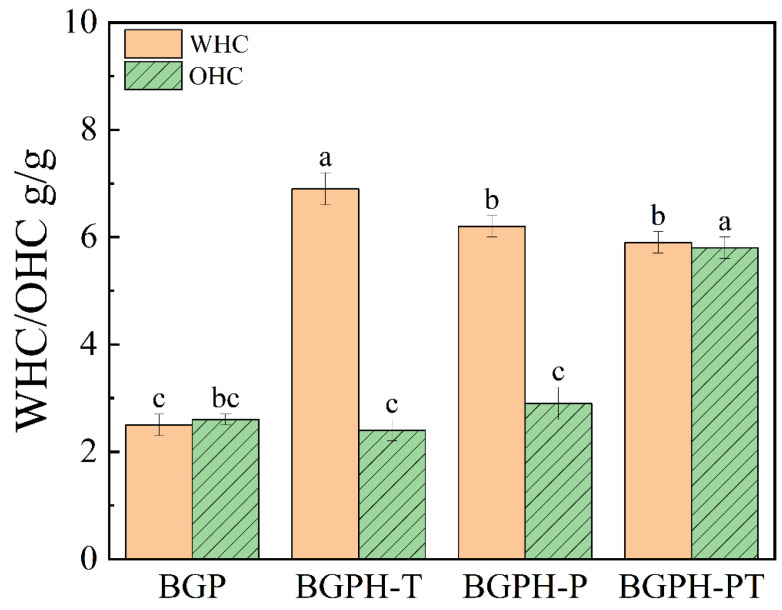
Water retention results and oil retention of BGP, BGPH-T, BGPH-P and BGPH-PT samples. Different letters marked in the same column indicate significant differences between groups (*p* < 0.05).

Water holding capacity (WHC) is a key indicator of a protein’s ability to bind water, reflecting the strength of its interaction with water molecules. In contrast, oil holding capacity (OHC) mainly represents the ability of non-polar side chains within the protein to interact with lipids [34], which directly influences the flavor, texture, and mouthfeel of protein-based products. These properties are crucial in the food industry, as they significantly enhance the physical characteristics of products and improve consumer experience [35]. Figure 7 presents the WHC and OHC measurements for black garlic protein (BGP) and its hydrolysates. Regarding WHC, the results showed the following order: BGPH-T > BGPH-P > BGPH-PT > BGP, indicating that enzymatic hydrolysis significantly improves WHC. During hydrolysis, proteins were cleaved into smaller peptide fragments, exposing more hydrophilic groups (e.g., amino and carboxyl groups), which facilitated stronger interactions with water molecules [36], thus increasing water retention. BGPH-T displayed the highest WHC, likely due to trypsin hydrolysis more efficiently exposing these hydrophilic groups. For OHC, the order is as follows: BGPH-PT > BGPH-P > BGP > BGPH-T. BGPH-PT exhibited the highest OHC, likely because dual enzymatic hydrolysis generated more hydrophobic fragments that bind more effectively with oil molecules, thereby enhancing oil retention [37]. In summary, BGPH-T had the highest WHC, while BGPH-PT showed the highest OHC. Overall, BGPH-PT demonstrated superior performance in both water and oil holding capacities, highlighting its exceptional functional properties.

#### 2.3.3. Results of Emulsifying Activity and Emulsifying Stability Measurement

**Figure 8:**
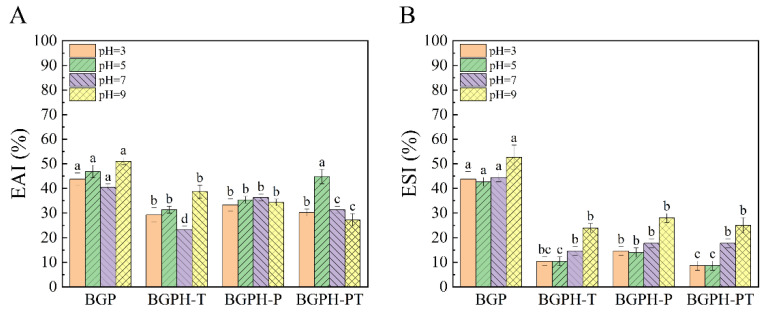
(**A**) EAI of BGP, BGPH-T, BGPH-P, and BGPH-PT samples. (**B**) ESI of BGP, BGPH-T, BGPH-P, and BGPH-PT samples. Different letters marked in the same column indicate significant differences between groups (*p* < 0.05).

Emulsifying activity (EAI) evaluates the ability of proteins to facilitate the formation of emulsions in oil-water mixtures, while emulsifying stability (ESI) indicates the ability of an emulsion to maintain stability over an extended period [38]. As illustrated in Figure 8, significant differences were observed in the emulsifying activity and stability of BGP, BGPH-T, BGPH-P, and BGPH-PT under various pH conditions (*p* < 0.05). Overall, the hydrolysates exhibited significantly lower emulsifying activity and stability when compared to the intact protein. This can be primarily attributed to enzymatic hydrolysis, which disrupts the intact protein structure into smaller peptide fragments, diminishing the interfacial adsorption capacity and surface activity, and thus making the formation of a stable oil-water interfacial film more difficult, ultimately reducing both emulsifying performance and stability [39]. The three hydrolysates (BGPH-T, BGPH-P, and BGPH-PT) exhibited different emulsifying activity and stability under various pH conditions. As shown in Figure 8A, BGPH-PT had the highest emulsifying activity at pH 5, while BGPH-T showed the lowest activity at pH 7. Notably, BGPH-P’s emulsifying activity remained relatively stable and was less affected by pH changes, whereas BGPH-T and BGPH-PT were more sensitive to pH fluctuations, showing greater variability in their emulsifying activity. A similar pattern was observed in emulsifying stability, as depicted in Figure 8B. BGPH-P displayed superior stability across all pH levels, while BGPH-T and BGPH-PT exhibited lower stability. This phenomenon can be caused by differences in the structural characteristics and charge distribution of the hydrolysates in response to pH changes. BGPH-P, characterized by its smaller particle size and higher Zeta potential, suggests the formation of more stable small peptides following enzymatic hydrolysis. These peptides maintain consistent interfacial adsorption capacity and membrane-forming ability across different pH conditions, leading to minimal changes in emulsifying activity and stability [40]. In contrast, BGPH-T and BGPH-PT seem more sensitive to pH variations, particularly under acidic or alkaline conditions. Significant changes in protein structure and charge distribution may occur, causing larger fluctuations in interfacial activity and emulsifying performance, resulting in greater pH sensitivity and reduced stability [41]. In conclusion, while enzymatic hydrolysis generally reduced the emulsifying activity and stability of the hydrolysates, BGPH-P showed the least sensitivity to pH changes, displaying better overall emulsifying activity and stability.

#### 2.3.4. Foaming Capacity and Foam Stability Measurements

**Figure 9:**
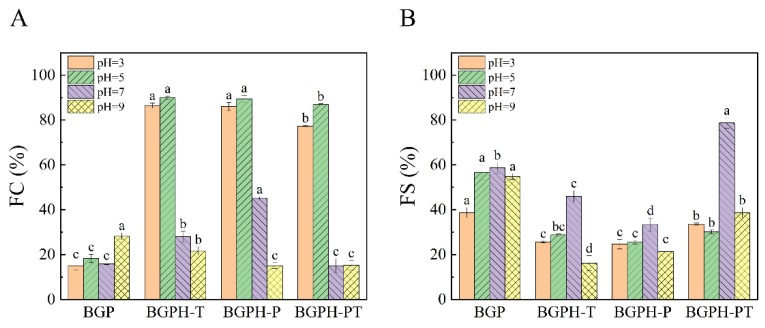
(**A**) Foaming activity of BGP, BGPH-T, BGPH-P, and BGPH-PT samples. (**B**) Foaming stability of BGP, BGPH-T, BGPH-P, and BGPH-PT samples. Different letters marked in the same column indicate significant differences between groups (*p* < 0.05).

Foaming capacity refers to the ability of a protein to reduce surface tension at the gas–liquid interface, promoting foam formation. Such a property is closely related to the surface activity of the protein [42], which is influenced by factors including charge state, molecular size, structure, and the pH of the solution. As illustrated in Figure 9, significant differences were observed in the foaming capacity and foam stability of BGP, BGPH-T, BGPH-P, and BGPH-PT (*p* < 0.05). Overall, enzymatic hydrolysis improved foaming capacity but reduced foam stability. Specifically, under acidic and mildly acidic conditions (pH 3 and pH 5), BGPH-T, BGPH-P, and BGPH-PT showed significantly higher foaming capacity than BGP (*p* < 0.05), as seen in Figure 9A. At pH 7, the samples exhibited notable differences, with BGPH-P having the highest foaming capacity. Interestingly, at pH 9, BGP exhibited higher foaming capacity than the hydrolysates. Overall, the foaming capacity followed the order: BGPH-P > BGPH-T > BGPH-PT > BGP. This increase in foaming capacity can be attributed to the breakdown of large protein molecules into smaller peptides during enzymatic hydrolysis, which enhances surface activity by enabling faster adsorption at the gas–liquid interface and reducing surface tension. However, at higher pH levels, especially under alkaline conditions, the peptide fragments tend to exhibit uniform charge distribution, leading to increased repulsion and decreased adsorption efficiency, thus reducing foaming capacity. Figure 9B shows that the foam stability of the hydrolysates decreased compared to that of BGP. Among the hydrolysates, BGPH-PT displayed the highest foam stability at pH 7, while BGPH-T was the lowest at pH 9. In general, foam stability followed the order: BGP > BGPH-PT > BGPH-T > BGPH-P. This decrease in foam stability can be attributed to the smaller peptide fragments produced by enzymatic hydrolysis, which lack the structural integrity of intact proteins, making it difficult to form a dense and stable interfacial film, thereby reducing foam stability [43]. In conclusion, BGP exhibited superior foaming capacity and stability at pH 9, making it suitable for applications in alkaline systems requiring stable foam. In contrast, BGPH-P, BGPH-T, and BGPH-PT demonstrated higher foaming capacity under acidic and mildly acidic conditions (pH 3 and pH 5), but had lower foam stability, suggesting their potential suitability for short-term foam applications.

### 2.4. Antioxidant Activity of Black Garlic Protein and Its Hydrolysates

Antioxidant activity refers to a substance’s ability to resist oxidation by inhibiting free radical formation or neutralizing existing free radicals. This ability is crucial for maintaining the health of living organisms [44]. DPPH and ABTS^+^ radical scavenging capacities are key indicators for evaluating the ability of antioxidant compounds to donate electrons or hydrogen atoms, thus reflecting their ability to convert free radicals into more stable species. The stronger this ability, the greater the potential to neutralize free radicals. Consequently, these indicators are critical in evaluating potential antioxidants in foods, pharmaceuticals, and cosmetics [45].

**Figure 10:**
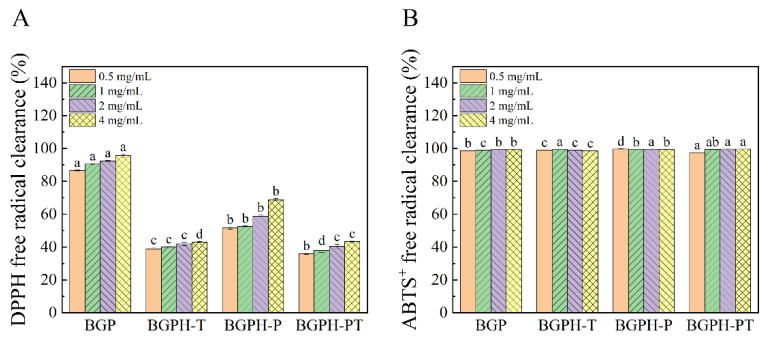
(**A**) DPPH free radical clearance of BGP, BGPH-T, BGPH-P, and BGPH-PT samples. (**B**) ABTS^+^ free radical clearance of BGP, BGPH-T, BGPH-P, and BGPH-PT samples. Different letters marked in the same column indicate significant differences between groups (*p* < 0.05).

As shown in Figure 10A, at the same concentration, the DPPH scavenging capacity followed the order: BGP > BGPH-P > BGPH-T > BGPH-PT. After enzymatic hydrolysis, the DPPH scavenging ability of the hydrolysates significantly decreased (*p* < 0.05). This suggests that the original protein structure of BGP may contain key antioxidant regions or active sites capable of effectively donating electrons or hydrogen atoms to neutralize free radicals. Among the three hydrolysates (BGPH-T, BGPH-P, and BGPH-PT), BGPH-P exhibited the highest DPPH radical scavenging capacity, reaching 68.81% at a concentration of 4 mg/mL. This result suggests that while pepsin partially hydrolyzed the protein, potentially compromising certain antioxidant regions [46], its antioxidant activity was preserved and even enhanced with increasing concentration. As shown in Figure 10B, BGP and its hydrolysates (BGPH-T, BGPH-P, and BGPH-PT) showed high antioxidant activity in the ABTS^+^ assay system (*p* < 0.05). This phenomenon may result from the higher sensitivity of the ABTS^+^ radical assay to hydrophilic antioxidants. Enzymatic hydrolysis exposes more hydrophilic groups within the protein [47], allowing them to interact more readily with water-soluble ABTS^+^ radicals and, thus, enhancing antioxidant activity. Notably, BGPH-P showed the highest antioxidant activity in the ABTS^+^ assay, with radical scavenging capacities exceeding 99% across different concentrations (0.5, 1, 2, and 4 mg/mL). This may be due to the exposure of more hydrophilic groups during pepsin hydrolysis of black garlic protein. These findings suggest that black garlic protein and its hydrolysates have strong potential in antioxidant applications and may serve as key materials for developing novel natural antioxidants. In conclusion, although the hydrolysates exhibited reduced DPPH scavenging capacity, all samples showed high antioxidant activity in the ABTS^+^ assay system. Overall, BGP showed the highest antioxidant activity, and among the three hydrolysates, BGPH-P exhibited the strongest antioxidant performance.

### 2.5. In Vitro Inhibitory Effects of Black Garlic Protein and Its Hydrolysates on Enzyme Activity

**Figure 11:**
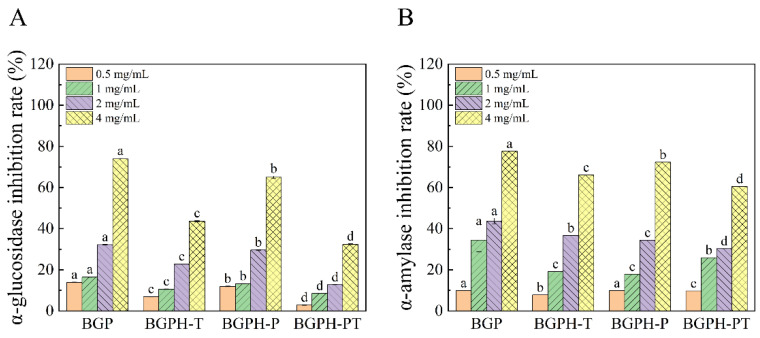
(**A**) α-glucosidase inhibition rate of BGP, BGPH-T, BGPH-P, and BGPH-PT samples. (**B**) α-amylase inhibition rate free radical clearance of BGP, BGPH-T, BGPH-P, and BGPH-PT samples. Different letters marked in the same column indicate significant differences between groups (*p* < 0.05).

α-Glucosidase and α-amylase are two key human enzymes critical for the digestion and absorption of carbohydrates [48]. α-Glucosidase, located in the brush border of the small intestine, hydrolyzes the non-reducing terminal α (1–4) bonds of oligosaccharides into monosaccharides, which are then absorbed by intestinal epithelial cells [49]. Thus, inhibiting α-glucosidase activity is an effective strategy to control glucose digestion and absorption in the small intestine, thereby reducing blood glucose levels. According to the experimental results displayed in Figure 11A, BGP exhibited a greater inhibitory effect on α-glucosidase than its hydrolysates. Specifically, the inhibitory effects of the samples were ranked as follows: BGP > BGPH-P > BGPH-T > BGPH-PT. At the concentration of 4 mg/mL, α-glucosidase inhibition rates by BGP, BGPH-T, BGPH-P, and BGPH-PT were 73.90%, 43.62%, 65%, and 32.32%, respectively. Both BGP and BGPH-P showed inhibition rates above 50%, indicating significant inhibitory effects. On the other hand, α-amylase, primarily produced by the salivary glands and pancreas, breaks down polysaccharides like starch into smaller sugar molecules such as glucose. This process is closely related to the regulation of blood glucose levels in the body. Inhibiting α-amylase activity slows starch breakdown, thereby preventing a rapid postprandial spike in blood glucose levels [50]. As shown in Figure 11B, at the same concentration (4 mg/mL), BGP, BGPH-T, BGPH-P, and BGPH-PT exhibited α-amylase inhibition rates above 50%, with values of 77.61%, 66.12%, 72.30%, and 60.52%, respectively. These results demonstrate the potential of black garlic protein and its hydrolysates in inhibiting α-amylase activity. Among the three hydrolysates, BGPH-P exhibited the strongest inhibitory effects on both α-glucosidase and α-amylase.

### 2.6. Activity (Antioxidant and Hypoglycemic) Comparison with Other Materials

Compared with other plant proteins, BGP exhibits significant advantages in both antioxidant and hypoglycemic activities (Table 4). At a concentration of 1 mg/mL, BGP demonstrates DPPH and ABTS^+^ radical scavenging activities exceeding 90%. In contrast, under the same conditions, the radical scavenging capacities of soy protein and quinoa protein remain below this threshold [51,52,53]. Furthermore, the enzymatic hydrolysates of BGP, including BGPH-T, BGPH-P, and BGPH-PT, consistently achieved ABTS^+^ radical scavenging activities above 95% across a range of concentrations (0.5, 1, 2, and 4 mg/mL). These values are significantly higher than those reported for quinoa protein hydrolysates, which remain below 90% [54]. In terms of hypoglycemic activity, Yang Xue et al. [55] observed that Lycium barbarum leaf protein exhibited α-glucosidase and α-amylase inhibitory rates below 20% at a protein concentration of 4 mg/mL. In contrast, at the same concentration (4 mg/mL), BGP and its hydrolysates (BGPH-T, BGPH-P, BGPH-PT) achieved α-glucosidase inhibitory rates of 73.90%, 43.62%, 65.00%, and 32.32%, respectively, and α-amylase inhibitory rates of 77.61%, 66.12%, 72.30%, and 60.52%, all of which were markedly higher than those of Lycium barbarum leaf protein. Additionally, wheat protein extract has been reported to exhibit an α-amylase inhibitory rate of 58.75% at a concentration of 5 mg/mL [56], while black sesame protein and its hydrolysates (<3 kDa fraction) demonstrated α-amylase inhibitory activity of 31.08% and α-glucosidase inhibitory activity of 57.25% [57]. These values are substantially lower than the α-glucosidase inhibitory rates observed for BGP and BGPH-P. In summary, BGP and its hydrolysates (BGPH-T, BGPH-P, BGPH-PT) display superior antioxidant and hypoglycemic activities, highlighting their potential as promising candidates for the development of functional foods with health-promoting properties.

## 3. Materials and Methods

### 3.1. Materials and Instruments

Materials: Heze Tianhong Fruits & Vegetables Co., Ltd Black garlic powder was obtained from Heze Tianhong Fruits & Vegetables Co., Ltd. (Heze, China). Anhydrous ethanol, sodium hydroxide, hydrochloric acid, and phosphoric acid (all analytical grade) were supplied by Tianjin Jiangtian Chemical Technology Co., Ltd. (Tianjin, China). Soluble starch and potassium persulfate were purchased from Shanghai Macklin Biochemical Co., Ltd. (Shanghai, China). Bovine serum albumin, pepsin, trypsin, and Coomassie Brilliant Blue G-250 were provided by Beijing Solarbio Science & Technology Co., Ltd. (Beijing, China). DPPH and ABTS^+^ were obtained from Shanghai Aladdin Biochemical Technology Co., Ltd. (Shanghai, China). α-Glucosidase, α-amylase, p-nitrophenyl-α-D-glucopyranoside, and 3,5-dinitrosalicylic acid were purchased from Hefei Xiyuan Biotechnology Co., Ltd. (Hefei, China).

Instruments: An electronic balance (Model: CN-LQC30002) was supplied by Kunshan Youkewei Electronic Technology Co., Ltd. (Kunshan, China). A high-speed desktop-refrigerated centrifuge (Model: TGL-16M) was purchased from Xiangyi Centrifuge Instrument Co., Ltd. (Changsha, China). A pH meter (Model: PHS-3E) was obtained from Shanghai Yidian Scientific Instrument Co., Ltd. (Shanghai, China). A digital magnetic stirrer (Model: SP-25) was provided by Hangzhou Mio Instrument Co., Ltd. (Hangzhou, China). A digital thermostatic water bath (Model: HH-4) was purchased from Jintan Shenglan Instrument Manufacturing Co., Ltd. (Jintan, China). A UV-Visible spectrophotometer (Model: T6 New Century) was obtained from Beijing Purkinje General Instrument Co., Ltd. (Beijing, China). A high-speed homogenizer (Model: RCD-1A) was supplied by Xicheng Xinrui Instrument Factory (Jintan District, China). A Fourier transform infrared spectrometer (Model: Nicolet iS20) was purchased from Thermo Fisher Scientific (Waltham, USA). A scanning electron microscope (Model: TESCAN MIRA LMS) was obtained from TESCAN (Brno, Czech Republic). A nanoparticle size and Zeta potential analyzer (Model: Nano ZS90) was provided by Malvern Zetasizer Nano (Malvern, UK).

### 3.2. Calculation of Black Garlic Protein Content and Yield

To accurately determine the protein content of black garlic, a standard curve for bovine serum albumin (BSA) was constructed. First, prepare a BSA standard solution at a concentration of 0.2 mg/mL. Pipette 0 μL, 100 μL, 200 μL, 300 μL, 400 μL, and 500 μL of the BSA standard solution into a series of pre-labeled centrifuge tubes. Then, add PBS buffer to each tube, ensuring a final volume of 500 μL. Next, add 5 mL of 1×G-250 dye solution to each tube, thoroughly shaking to mix. Allow the tubes to stand for 3 min to complete the staining reaction. The absorbance (OD value) of each standard solution is measured at 595 nm using a spectrophotometer. Finally, plot the BSA concentration on the x-axis and the corresponding OD value on the y-axis to establish the standard curve and its regression equation [58].

Pipette 0.5 mL of the sample solution (prepared by dissolving 0.1 g of freeze-dried black garlic in distilled water and adjusting the final volume to 20 mL). Add 5 mL of Coomassie Brilliant Blue G-250 solution and mix thoroughly to ensure full interaction between the dye and the sample solution. Allow the mixture to stand for 3 min to complete the staining reaction. Subsequently, measure the absorbance of the sample solution at 595 nm using a spectrophotometer, following the same procedure as the standard curve preparation, with distilled water as the blank control. The black garlic protein concentration is calculated from the standard curve. Finally, the black garlic protein content is calculated from the following Equation (1).

(1) $$The\;protein\;content\;in\;the\;sample\left(g/100\;g\right)=\frac{\left(C-C_{0}\right)\times V}{m\times 1000}\times 100$$

in which *C* denotes the protein concentration derived from the standard curve (mg/mL), *C*_0_ represents the protein concentration of the blank test (mg/mL), *V* is the final volume of the sample solution (mL), and *m* is the mass of the sample (g).

Firstly, the black garlic protein is extracted via alkali extraction and acid precipitation from black garlic powder with 1 g. Then, the obtained black garlic protein is freeze-dried, collected, and weighed. The mass of the freeze-dried black garlic protein is substituted into Equation (2) to obtain its yield.

(2) $$Yield\left(\%\right)=\frac{M_{1}}{M_{2}}\times 100$$

in which *M*_1_ represents the mass of the black garlic protein extract (g) and *M*_2_ represents the mass of the black garlic powder (g).

### 3.3. Optimal Extraction Process of Black Garlic Protein

#### 3.3.1. Determination of the Isoelectric Point

To accurately determine the isoelectric point of black garlic protein, protein yield was served as the key indicator, and the following steps were followed: First, an appropriate amount of black garlic powder was dissolved in distilled water at a mass-to-volume ratio of 1:40, and the pH was adjusted to 10. The solution was extracted at 40 °C for 30 min and then centrifuged. The extract was evenly divided into seven portions, and the pH of each was adjusted to 2.8, 3.1, 3.4, 3.7, 4.0, 4.3, and 4.6, respectively. The solutions were left to stand for 30 min to ensure pH equilibrium. Afterwards, the samples were centrifuged at 10,000 rpm for a duration of 15 min at a temperature of 4 °C. The precipitates were gathered and subjected to freeze-drying in order to obtain the samples for further analysis. Finally, the protein yield at each pH was calculated and compared to accurately determine the isoelectric point of black garlic protein.

#### 3.3.2. Single-Factor Experimental Design and Implementation

In this experiment, protein yield served as the core indicator to investigate the specific effects of four key factors—solid-to-liquid ratio, extraction time, extraction temperature, and pH—on black garlic protein yield. A single-factor experimental design was adopted, and black garlic protein was extracted via alkali extraction followed by acid precipitation. The pH during the acid precipitation stage was fixed at 3.1 (the isoelectric point of black garlic protein, determined in Section 3.3.1).

During alkali extraction, three factors were held constant while varying the investigated factor. The specific experimental steps were as follows: First, with the solid-to-liquid ratio set to 1 g:30 mL, extraction temperature at 30 °C, and pH adjusted to 8 (by adding alkali), extraction times of 60, 80, 100, 120, and 140 min were applied to observe their effect on protein yield. Second, with extraction time fixed at 100 min, temperature at 30 °C, and pH at 8, the solid-to-liquid ratio was set to 1 g:30 mL, 1 g:40 mL, 1 g:50 mL, 1 g:60 mL, and 1 g:70 mL to investigate its influence on protein yield. Third, with extraction time fixed at 100 min, solid-to-liquid ratio at 1 g:50 mL, and pH at 8, extraction temperatures of 30, 40, 50, 60, and 70 °C were tested to analyze their effect on protein yield. Finally, with extraction time fixed at 100 min, solid-to-liquid ratio at 1 g:50 mL, and extraction temperature at 30 °C, pH values of 7, 8, 9, 10, and 11 were applied by adjusting alkali amounts for the alkali extraction. Subsequently, during the acid precipitation stage, the pH was adjusted to 3.1 (the isoelectric point of black garlic protein) to evaluate the effect of different alkali extraction pH values on protein yield.

#### 3.3.3. Optimization of Extraction Conditions for Black Garlic Protein Using the Response Surface Methodology

This study focused on protein yield as the primary indicator, aiming to systematically investigate the effects of four critical factors—extraction time (A), solid-to-liquid ratio (B), extraction temperature (C), and pH (D)—on the protein yield of black garlic (Table 5). A single-factor experimental design was employed, using the alkali-solubilization and acid-precipitation method for protein extraction. The pH during the acid-precipitation stage was fixed at 3.1, corresponding to the isoelectric point of black garlic protein, as determined in advance using the method outlined in Section 3.3.1.

The experimental design was carefully constructed using Design-Expert 12 software, resulting in 29 experimental conditions. To ensure data reliability and accuracy, 7 replicate experiments were included for further validation. ANOVA (analysis of variance) was applied during the data analysis phase to establish a regression model that included the main factors (A, B, C, D), all possible interactions (AB, AC, AD, BC, BD, CD), and quadratic terms (A^2^, B^2^, C^2^, D^2^).

### 3.4. Preparation of Black Garlic Protein Hydrolysates

Black garlic protein was prepared under optimal extraction conditions and subsequently freeze-dried. The freeze-dried black garlic protein was treated with enzymatic hydrolysis using pepsin, trypsin, and a combination of both enzymes. The hydrolysis conditions were as follows: pepsin was added at 5% (*w*/*w*) and hydrolysis was performed at pH 2.0; or trypsin was added at 5% (*w*/*w*) and hydrolysis was conducted at pH 8.0; or pepsin was first added at 5% (*w*/*w*) at pH 2.0, followed by trypsin at 5% (*w*/*w*) at pH 8.0. All enzymatic reactions were carried out at 37 °C for 3 h. After hydrolysis, the enzymes were inactivated by placing the mixture in boiling water for 10 min [59]. The solution was then centrifuged at 10,000 rpm for 15 min at 4 °C, and the supernatant was collected and freeze-dried for storage. Ultimately, four samples were obtained: crude black garlic protein (BGP), pepsin hydrolysate (BGPH-P), trypsin hydrolysate (BGPH-T), and dual enzyme hydrolysate (BGPH-PT) derived from sequential hydrolysis with pepsin and trypsin.

### 3.5. Structural Characterization of Black Garlic Protein and Its Hydrolysates

#### 3.5.1. Measurement of Particle Size and Zeta Potential

The particle size and Zeta potential of the samples (BGP, BGPH-T, BGPH-P, and BGPH-PT) were measured following the method of Nesterenko et al. [60], with minor adjustments. Each sample powder was dissolved in phosphate buffer (10 mmol/L, pH 7.4) at a concentration of 1% (*w*/*v*) to create sample mixtures. The sample mixtures were then added dropwise into a cuvette, adjusting the refractive index to 20–40%. Subsequently, the average particle size distribution and Zeta potential of each sample were measured at 25 °C using a particle size and potential analyzer. All measurements were performed in triplicate to ensure accuracy. During the measurements, a refractive index of 1.450 was used for the dispersant (protein and hydrolysates), while a refractive index of 1.331 was used for the continuous phase.

#### 3.5.2. Scanning Electron Microscopy Observation

A small amount of BGP, BGPH-T, BGPH-P, and BGPH-PT samples were placed onto conductive adhesive and coated with gold using a Quorum SC7620 sputter coater at a sputtering current of 10 mA, with the duration adjusted based on the sample and testing requirements. The samples were subsequently examined using a scanning electron microscope for morphology imaging and energy-dispersive spectroscopy (EDS) mapping. An acceleration voltage of 3 kV was used for morphology imaging, and 15 kV for EDS mapping, with an SE2 secondary electron detector employed for detection [61].

#### 3.5.3. Analysis by Fourier Transform Infrared Spectroscopy

Samples of BGP, BGPH-T, BGPH-P, and BGPH-PT (20 mg each) were weighed accurately and placed into a dry mortar. Subsequently, 200 mg of dry potassium bromide (KBr) powder was introduced. After thorough mixing, the mixture was pressed into thin pellets under a pressure of 4 tons using a tablet press. The infrared spectra of each sample were obtained by scanning in the wavenumber range of 400/600 to 4000 cm^−1^ (32 scans) using a spectrometer. All samples were tested under identical conditions during spectral analysis. Baseline correction and peak identification were performed during data processing [62].

### 3.6. Functional Properties of Black Garlic Protein and Its Hydrolysates

#### 3.6.1. Solubility Measurement

We weighed 0.4 g of BGP, BGPH-T, BGPH-P, and BGPH-PT samples and dissolved each in 80 mL of distilled water to prepare 5 mg/mL solutions. Each sample was divided into four portions, and the pH was adjusted to 3, 5, 7, and 9 using 1.0 mol/L NaOH or 1.0 mol/L HCl. After pH adjustment, the solutions were stirred for 2 h at 25 °C to ensure complete dissolution, followed by centrifugation at 8000× *g* rpm for 10 min. The soluble protein content at different pH levels was determined using the Coomassie Brilliant Blue assay at 595 nm. Protein content was calculated using Equation (1), and solubility was determined using Equation (3) [63]:

(3) $$Soulubility\left(\%\right)=\frac{p_{2}}{p_{1}}\times 100\%$$

in which *p*_2_ denotes the protein concentration in the supernatant, while *p*_1_ refers to the total protein concentration.

#### 3.6.2. Water-Holding Capacity Measurement

We accurately weighed 0.8 g of BGP, BGPH-T, BGPH-P, and BGPH-PT samples, and dissolved each in 40 mL distilled water to prepare 20 mg/mL solutions. Each sample solution was divided into four portions, and the pH was adjusted to 3, 5, 7, and 9 using 1.0 mol/L NaOH or 1.0 mol/L HCl. The solutions were mixed using a vortex for 6 min and incubated at 30 °C for 30 min in a thermostatic chamber. Afterwards, the dispersed solutions were centrifuged at 5000× g rpm for 20 min, and the supernatant was gently poured off to avoid disturbing the residue. The centrifuge tubes were inverted and left for 20 min to drain any residual water. Finally, the total mass of the centrifuge tube and precipitate was measured [64]. The percentage of water-binding capacity was calculated using Equation (4):

(4) $$WHC=\frac{x-y}{z}$$

in which *x* represents the mass of the polypeptide and the test tube after water absorption, *y* is the mass of the polypeptide and the test tube before water absorption, and *z* is the mass of the polypeptide before water absorption.

#### 3.6.3. Oil-Holding Capacity Measurement

We accurately weighed 0.2 g of each of the BGP, BGPH-T, BGPH-P, and BGPH-PT samples, and added 10 mL of soybean oil to each. The samples were vortexed for 6 min until they were saturated with oil, and then we let them stand for 30 min. They were then centrifuged at 5000× *g* rpm for 20 min and the supernatant was removed. The centrifuge tubes were then inverted and left for 20 min to drain any residual oil. Finally, we weighed the total mass of the centrifuge tubes and precipitates [65]. The percentage of oil-binding capacity was calculated using Equation (5):

(5) $$OHC=\frac{x-y}{z}$$

where *x* represents the mass of the polypeptide and the test tube after oil absorption, *y* is the mass of the polypeptide and the test tube before oil absorption, and *z* is the mass of the polypeptide before oil absorption.

#### 3.6.4. Emulsifying Activity and Stability Measurement

In this study, the emulsifying activity (EAI) and emulsifying stability (ESI) of each sample were measured following the method of Malik et al. [66], with some modifications. The experimental steps were as follows: First, 0.4 g of each BGP, BGPH-T, BGPH-P, and BGPH-PT sample was weighed and dissolved in 20 mL distilled water to prepare 20 mg/mL solutions. Each solution was divided into four 5 mL portions, and the pH was adjusted to 3, 5, 7, and 9 using 1.0 mol/L NaOH or 1.0 mol/L HCl. Subsequently, 5 mL of soybean oil (1:1 ratio) was added, and the mixtures were homogenized at 12,000 rpm for 2.5 min at room temperature. After homogenization, the emulsions were centrifuged at 3000× *g* rpm for 10 min to separate the emulsion layer from the remaining contents. Emulsification performance was analyzed by measuring the height of the emulsion layer and the total content height. To evaluate emulsifying stability, the emulsions were heated at 80 °C for 30 min, cooled to room temperature, and centrifuged at 2000× *g* rpm for 5 min. The EAI and ESI were calculated using Equations (6) and (7), respectively.

(6) $$EAI=\frac{The\;emulsified\;layer’s\;height}{Total\;content\;height\;in\;the\;tube}\times 100$$

(7) $$ESI=\frac{The\;emulsified\;layer’s\;height\;after\;heating}{Total\;content\;height\;in\;the\;tube}\times 100$$

#### 3.6.5. Measurement of Foaming Capacity and Foaming Stability

In this study, foaming capacity (FC) and foaming stability (FS) of each sample were measured following the method of Feng et al. [67], with some adjustments. The steps are as follows: We accurately weighed 0.4 g of each sample (BGP, BGPH-T, BGPH-P, and BGPH-PT) and dissolved it in 40 mL distilled water to prepare 10 mg/mL solutions. Each solution was divided into four 10 mL portions, and the pH was adjusted to 3, 5, 7, and 9 using 1.0 mol/L NaOH or HCl. The solutions were then homogenized at 12,000 rpm for 2.5 min using a high-speed homogenizer. After homogenization, the foam volume was immediately measured and recorded using a graduated cylinder. The foam was left to stand at 25 °C for 30 min, and the remaining foam volume was measured again. Finally, FC and FS were calculated using Equations (8) and (9) to quantify the foaming performance and stability of each sample.

(8) $$FC\left(\%\right)=\frac{V_{1}}{V_{0}}\times 100$$

(9) $$FS\left(\%\right)=\frac{V_{2}}{V_{1}}\times 100$$

in which *V*_0_ represents the initial volume of the sample solution, *V*_1_ represents the foam volume after 2.5 min of homogenization, and *V*_2_ represents the foam volume after 30 min of standing.

### 3.7. Measurement of Antioxidant Activity of Black Garlic Protein and Its Hydrolysates

#### 3.7.1. DPPH Radical Scavenging Activity

DPPH radical scavenging activity was determined according to the method of Renn et al. [68], with some modifications. A total of 0.0197 g DPPH was dissolved in 95% ethanol in a 250 mL volumetric flask to prepare a 0.2 mmol/L working solution. BGP, BGPH-T, BGPH-P, and BGPH-PT samples were prepared at different concentrations (0.5, 1.0, 2.0, and 4.0 mg/mL). Subsequently, 2 mL of the DPPH solution was combined with 2 mL of each sample solution, thoroughly vortexed, and allowed to react in the dark at room temperature for 30 min. After the reaction, the absorbance was measured at a specific wavelength using a spectrophotometer. Finally, the radical scavenging activity was calculated using Equation (10) to quantify the antioxidant capacity of each sample.

(10) $$DPPH\;Radical\;Scavenging\;Rate(\%)=\left(1-\frac{A_{1}-A_{2}}{A_{0}}\right)\times 100$$

in which *A*_0_ represents 2 mL DPPH + 2 mL 95% ethanol, *A*_1_ represents 2 mL sample + 2 mL 95% ethanol, *A*_2_ represents 2 mL sample + 2 mL DPPH.

#### 3.7.2. ABTS^+^ Radical Scavenging Activity

The ABTS^+^ scavenging activity was measured following the method of Wang et al. [69], with some modifications. A total of 0.192 g ABTS^+^ and 0.033 g potassium persulfate were dissolved separately in 50 mL of distilled water, mixed thoroughly, and kept in the dark at 25 °C for 12−16 h to prepare the ABTS^+^ stock solution. The stock solution was diluted with distilled water to an absorbance of 0.7 ± 0.02 at 734 nm, serving as the ABTS^+^ working solution. Next, BGP, BGPH-T, BGPH-P, and BGPH-PT samples were prepared at different concentrations (0.5, 1.0, 2.0, and 4.0 mg/mL). Then, 0.5 mL of each sample was added to each test tube, followed by 5 mL of ABTS^+^ working solution, and the mixtures were shaken. A control group (*A_0_*: 0.5 mL ethanol solution + 5 mL working solution) and a blank group (*A_2_*: 0.5 mL sample solution + 5 mL distilled water) were also prepared. The test tubes were kept in the dark at 25 °C for 10 min, and the absorbance at 734 nm was measured three times to ensure accuracy. Finally, the radical scavenging activity was calculated using Equation (11) to quantify the antioxidant capacity of each sample.

(11) $${ABTS}^{+}Radical\;Scavenging\;Rate\left(\%\right)=\left(1-\frac{A_{1}-A_{2}}{A_{0}}\right)\times 100$$

in which *A*_0_ represents 0.5 mL distilled water and 5 mL ABTS^+^ solution, *A*_1_ represents 0.5 mL sample and 5 mL ABTS^+^ solution, and *A*_2_ 0.5 mL sample and 5 mL distilled water.

### 3.8. In Vitro Enzyme Inhibition Assay of Black Garlic Protein and Its Hydrolysates

#### 3.8.1. α-Glucosidase Inhibition Assay

The assay was performed with slight modifications following the method of Zhang et al. [70]. BGP, BGPH-T, BGPH-P, and BGPH-PT samples were prepared at concentrations of 0.5, 1, 2, and 4 mg/mL in distilled water. Next, 200 μL of each sample solution was mixed with 200 μL of α-glucosidase solution (0.5 U/mL) and incubated at 37 °C in a water bath for 10 min. Afterward, 200 μL of 4-nitrophenyl α-D-glucopyranoside (5 mmol/L) was added and incubated at 37 °C for 30 min to complete the enzymatic reaction. The reaction was terminated by adding 200 μL of Na_2_CO_3_ solution (1 mmol/L), and the total volume was adjusted with 3 mL of distilled water. The absorbance of the reaction product was measured at 405 nm to determine the amount of product formed. The experiment was repeated three times to ensure data reliability. Finally, the α-glucosidase inhibition rate of each sample was calculated using Equation (12).

(12) $$\alpha -Glucosidase\;Inhibition\;Rate=1-\frac{A_{1}-A_{2}}{A_{0}}\times 100$$

where *A*_0_ represents the absorbance value when distilled water is used instead of the sample solution, *A*_1_ represents the absorbance value of the sample solution with reagents, and *A*_2_ represents the absorbance value of the sample solution itself, with distilled water used in place of the α-glucosidase solution.

#### 3.8.2. α-Amylase Inhibition Assay

The assay was performed with slight modifications following Apostolidis E et al. [71]. BGP, BGPH-T, BGPH-P, and BGPH-PT samples were prepared at concentrations of 0.5, 1, 2, and 4 mg/mL in distilled water. Next, 0.2 mL of each sample solution was mixed with 0.2 mL of α-amylase solution (0.5 U/mL) and incubated at 37 °C in a water bath for 10 min. After incubation, 0.2 mL of 1% soluble starch solution was added, and the mixture was incubated for another 30 min in the water bath. Next, 0.4 mL of 0.1% 3,5-dinitrosalicylic acid (DNS) reagent was added, heated in a boiling water bath for 10 min, followed by 3 mL distilled water to adjust the total volume. Finally, the absorbance was measured at 540 nm. The experiment was repeated three times to ensure data accuracy. The α-amylase inhibition rate was obtained from the following Equation (13):

(13) $$\alpha -Amylase\;Inhibition\;Rate=1-\frac{A_{1}-A_{2}}{A_{0}}\times 100\;$$

in which *A*_0_ represents the absorbance value when distilled water is used instead of the sample solution, *A*_1_ represents the absorbance value of the sample solution with reagents, and *A*_2_ represents the absorbance value of the sample solution itself, with distilled water used in place of the α-amylase solution.

### 3.9. Statistical Analysis

Data were analyzed using IBM SPSS Statistics 25.0, including descriptive statistics, variance (ANOVA), and regression analysis, to evaluate the effects of various factors on black garlic protein extraction yield and the properties of its hydrolysates. Significance was set at *p* < 0.05 and *p* < 0.01 to determine statistical differences. Graphs were created using Origin 2023.0 to display experimental results, providing strong support for scientific conclusions. To ensure the accuracy of the experimental results, all the aforementioned steps were repeated three times.

## 4. Conclusions

This study presents the first systematic investigation and optimization of the extraction process of black garlic protein (BGP) using an alkaline-soluble acid-precipitation method. The optimal extraction conditions were: a solid-to-liquid ratio of 1:50, extraction time of 100 min, temperature of 30 °C, and pH of 9.0. Under these conditions, the actual yield of BGP was 12.10% ± 0.21%, consistent with the predicted value. Structural, physicochemical, and bioactivity analyses of BGP and its hydrolysates (BGPH-T, BGPH-P, and BGPH-PT) revealed that BGP exhibited good emulsifying properties, antioxidant activity, and hypoglycemic activity. The enzymatic hydrolysates showed reduced particle size and increased absolute Zeta potential, with BGPH-P prepared using pepsin having the smallest particle size (188.57 ± 1.93 nm) and the highest absolute Zeta potential (−29.93 ± 0.42 mV), enhancing solubility and dispersion in water. SEM images indicated enhanced structural dispersion and surface pore formation in the protein after enzymatic hydrolysis, likely redistributing hydrophobic and hydrophilic groups and affecting functional properties like solubility, water-holding capacity, and oil-holding capacity. While hydrolysates showed superior water-holding and oil-holding capacities (BGPH-PT being the highest), unhydrolyzed BGP excelled in emulsifying activity and emulsion stability. In antioxidant evaluations, BGP demonstrated higher DPPH radical scavenging activity than its hydrolysates, with increasing activity across sample concentrations. ABTS^+^ radical scavenging activities were comparable among BGP and its hydrolysates. In vitro enzyme inhibition assays showed strong inhibitory effects on α-glucosidase and α-amylase for both BGP and its hydrolysates at 4 mg/mL, indicating notable hypoglycemic activity. This study provides scientific evidence for the potential applications of black garlic protein and its hydrolysates in food, nutraceutical, and pharmaceutical industries, promoting the expansion of black garlic products.

## Figures and Tables

**Table 1:** Experimental design and results of response surface optimization of black garlic protein preparation technology.

Test Number	A: Extraction Time (min)	B: Solid–Liquid Ratio (g/mL)	C: Extraction Temperature (°C)	D: pH	Yield (%)
1	120	50	30	10	11.35
2	100	50	20	10	10.43
3	80	60	30	9	11.58
4	80	40	30	9	10.72
5	120	50	30	8	10.69
6	100	60	30	10	10.84
7	100	40	30	8	10.62
8	100	40	30	10	10.83
9	120	50	20	9	11.06
10	100	60	20	9	10.89
11	120	50	40	9	11.47
12	100	50	30	9	12.25
13	100	40	40	9	10.95
14	80	50	20	9	11.37
15	80	50	40	9	10.92
16	80	50	30	8	11.28
17	100	40	20	9	11.06
18	100	50	20	8	11.32
19	120	60	30	9	11.05
20	80	50	30	10	11.11
21	100	50	30	9	12.18
22	100	50	30	9	12.19
23	100	50	30	9	12.18
24	100	60	30	8	11.05
25	100	50	40	10	11.91
26	100	60	40	9	11.52
27	120	40	30	9	11.79
28	100	50	30	9	12.4
29	100	50	40	8	10.24

**Table 2:** Regression model variance analysis of protein extraction rate of black garlic.

Source	Sum of Squares	df	Mean Square	F-Value	*p*-Value	Significance
Model	908.98	14	64.93	27.72	<0.0001	**
A-Time	1.54	1	1.54	0.6579	0.4309	
B-Solid–liquid ratio	7.68	1	7.68	3.28	0.0917	
C-Temperature	6.45	1	6.45	2.76	0.1192	*
D-pH	13.44	1	13.44	5.74	0.0311	*
AB	64	1	64	27.32	0.0001	**
AC	18.49	1	18.49	7.89	0.0139	*
AD	17.22	1	17.22	7.35	0.0169	*
BC	13.69	1	13.69	5.84	0.0298	*
BD	4.41	1	4.41	1.88	0.1916	
CD	163.84	1	163.84	69.95	<0.0001	**
A^2^	107.27	1	107.27	45.8	<0.0001	**
B^2^	228.03	1	228.03	97.36	<0.0001	**
C^2^	205.54	1	205.54	87.76	<0.0001	**
D^2^	361.63	1	361.63	154.4	<0.0001	**
Residual	32.79	14	2.34			
Lack of Fit	29.25	10	2.93	3.31	0.1303	not significant
Pure Error	3.54	4	0.885			
Cor Total	941.77	28				

Note: * indicates significant effect (*p* < 0.05); ** indicates very significant effect (*p* < 0.01).

**Table 3:** Analysis of mean particle size and Zeta potential of black garlic protein and its hydrolysates.

Sample	Mean Particle Size (nm)	Zeta Potential (mV)
BGP	200.07 ± 2.86 ^a^	−21.60 ± 1.85 ^a^
BGPH-T	193.73 ± 0.69 ^b^	−26.73 ± 1.00 ^b^
BGPH-P	188.57 ± 1.93 ^c^	−29.93 ± 0.42 ^c^
BGPH-PT	191.20 ± 0.65 ^bc^	−29.73 ± 0.56 ^c^

Note: Different letters indicate significant differences (*p* < 0.05).

**Table 4:** Comparison of the activity of black garlic protein and its hydrolysate with other materials.

Plant	Protein/Peptide	Concentration	DPPH Scavenging Rate	ABTS^+^ Scavenging Rate	α-Glucosidase Inhibition Rate	α-Amylase Inhibition Rate	Reference
Black Garlic	BGP	4 mg/mL	91.53%	98.92%	73.90%	77.61%	/
	BGPH-T	4 mg/mL	40.11%	99.55%	43.62%	66.12%	/
	BGPH-P	4 mg/mL	52.54%	99.26%	65.00%	72.30%	/
	BGPH-PT	4 mg/mL	43.05%	99.43%	32.32%	60.52%	/
Soybean	Protein/its hydrolysates	10 mg/mL	<20%	43%	/	/	[51,52]
Quinoa	Protein/its hydrolysates	10 mg/mL	<50%	<90%	44.79%	/	[53,54]
Lycium Leaf	Protein/its hydrolysates	4 mg/mL	/	/	<20%	<20%	[55]
Wheat	Protein/its hydrolysates	5 mg/mL	/	/	/	58.75%	[56]
Black Sesame	Protein/its hydrolysates	10 mg/mL	/	/	57.25%	31.08%	[57]

**Table 5:** Response surface experimental factors and levels.

Levels	A Extraction Time (min)	B Solid-to-Liquid Ratio (g/mL)	C Extraction Temperature (°C)	D pH
−1	80	40	20	8
0	100	50	30	9
1	120	60	40	10

## Data Availability

The data are available from the corresponding author upon suitable request.
